# Chitinase-3-like 1-protein in CSF: a novel biomarker for progression in patients with multiple sclerosis

**DOI:** 10.1007/s10072-023-06764-2

**Published:** 2023-03-29

**Authors:** Foraysa Talaat, Sahar Abdelatty, Christine Ragaie, Ahmed Dahshan

**Affiliations:** 1 Neurology Department, Cairo University, Giza, Egypt; 2 Clinical Pathology Department, Cairo University, Giza, Egypt

**Keywords:** Multiple sclerosis, CSF CHI3L1, EDSS, Cognitive impairment, Disability

## Abstract

**Background:**

Chitinase -3-like 1-protein (CHI3L1) is a glycoside secreted by monocytes, microglia, and activated astrocytes. Its distribution in inflammatory lesions denotes its role in astrocytic response to modulate CNS inflammation. In multiple sclerosis (MS), CHI3L1 levels have been found to be influenced by disease severity, activity, and progression. We aimed to measure CSF level of CHI3L1 in patients with MS and correlate its level with disability measures for a possible role as a biomarker for disease progression.

**Methods:**

Fifty-two MS patients (30 relapsing-remitting MS and 22 progressive MS) and thirty-five age and sex-matched healthy controls were included. They all underwent full clinical assessment (including disability and cognitive scales), radiological assessment, and CSF level of CHI3L1.

**Results:**

Patients with MS had higher CSF level of CHI3L1 than controls. Patients with progressive forms had higher levels than relapsing forms. There were positive correlations between disease duration, number of attacks, total EDSS, and CSF level of CHI3L1. Patients who had higher level of CSF CHI3L1 showed worse performance in MMSE and BICAMS and more lesions in T2 MRI brain. A cut off value of 154 ng/mL was found between patients with RRMS and PMS patients.

**Conclusion:**

CHI3L1 can be considered as a biomarker of disease progression. CHI3L1 level increases in progressive MS more than RRMS. Also, high CSF level of CHI3L1 was associated with more disability including motor, cognitive, and radiological aspects.

## Introduction

Multiple sclerosis (MS) is a complex autoimmune disease that affects the central nervous system (CNS), which leads to chronic inflammatory demyelination and neurodegeneration. Despite advances in diagnostic and therapeutic approaches, MS continues to pose significant challenges due to its heterogeneous nature in terms of clinical presentation, radiological and histopathological changes, and therapy response. Lesion load in the central nervous system, as detected by magnetic resonance imaging (MRI), and clinical characteristics such as relapse rate and disability progression, has long been important factors in the diagnosis and prognosis of multiple sclerosis. However, recent research has highlighted the importance of atrophy and cortical lesions, instead of focusing on the amount of lesions in white matter and relapse rates, in predicting progression and cognitive impairment in MS [1]. Unfortunately, in most cases, these available parameters used to assess different disease aspects, especially progression, are still subjective. Therefore, there is an urgent need to identify specific biomarkers that can aid in the diagnosis, prognosis, and monitoring of MS patients.

Recent studies have highlighted the potential of chitinase-3-like-1 protein (CHI3L1), also known as YKL-40, to serve as a promising biomarker for MS progression. CHI3L1 is a glycoprotein secreted by monocytes, microglia, and activated astrocytes and has been implicated in various inflammatory diseases, including MS, where it is believed to play a significant role in modulating CNS inflammation. CHI3L1 can be detected in both cerebrospinal fluid (CSF) and serum, making it a potentially accessible biomarker for MS patients. CHI3L1 is also produced by a broad spectrum of cells including macrophages, neutrophils, chondrocytes, synovial cells, osteoblasts, and vascular smooth muscle cells. Its expression is upregulated in response to inflammatory stimuli, and it has been implicated in various chronic inflammatory diseases [2]. Studies have shown that CHI3L1 levels in CSF are elevated in MS patients compared to healthy controls, and that these levels correlate with disease severity and progression. For instance, a study reported significantly elevated CHI3L1 levels in cerebrospinal fluid (CSF) of MS patients as compared to healthy individuals, and that higher levels of CHI3L1 were linked with increased disability and disease progression [3]. Another study found that elevated CHI3L1 levels in serum were associated with a higher risk of relapse in MS patients [4]. Additionally, CHI3L1 has been linked with the neurodegeneration that is a hallmark of progressive MS, indicating its potential as a valuable biomarker for tracking disease progression and predicting therapy response [5].

Nevertheless, additional research is required to thoroughly comprehend the role of CHI3L1 in MS pathogenesis, and its potential as a biomarker. Specifically, longitudinal studies that measure CHI3L1 levels over time in MS patients are necessary to evaluate if alterations in CHI3L1 levels correlate with disease progression or treatment response. Additionally, further studies are needed to investigate the underlying mechanisms by which CHI3L1 contributes to MS pathogenesis, which could lead to the development of novel therapies for this debilitating disease.

In this context, the aim of our study is to measure CSF level of CHI3L1 in MS patients and correlate its level with different aspects of disability including EDSS, cognition, and radiological aspects and if it could be used as an objective biomarker for disease progression. We hope to gain insights into the underlying mechanisms of MS progression and identify novel targets for therapeutic intervention.

## Methods

### Subjects

**Fig. 1:**
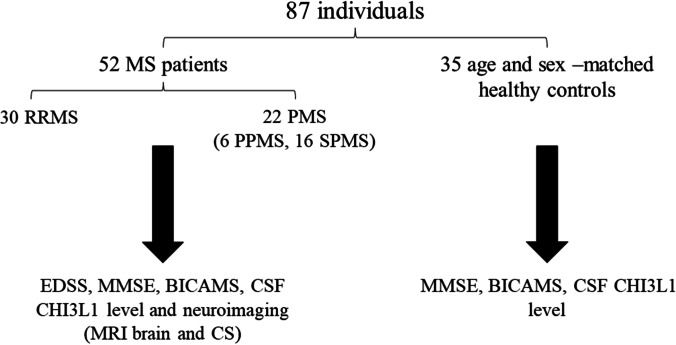
Study groups and interventions

This was a case-control study conducted on 87 Egyptian individuals divided into two groups. The patient group (52 MS patients) who were diagnosed according to revised McDonald Criteria 2017 [6], and control group (35 healthy individuals) (Fig. 1).

The study included patients aged between 18 and 60 years of both sexes who were relapse free at least 1 month before CSF sampling. Also, patients were off steroids at least 1 month before CSF sampling. We excluded patients and controls whose criteria are mentioned in Table 1.

**Table 1:** Exclusion criteria

Patients	Controls
Patients < 18 and > 60 years in age. Patients who were diagnosed as CIS or RIS. Patients with any disease other than MS. Patients who had autoimmune disorder other than MS. Patients who had neoplasm. Patients who had diabetes mellitus. Patients who had depression for proper assessment of cognition.	Control individuals < 18 and > 60 years in age Individuals who had neurological disorders. Individuals who had autoimmune disorders. Healthy control individuals who had neoplasm. Healthy control individuals who had diabetes mellitus.

### Methods

All patients underwent clinical assessment in the form of full history taking, general medical examination and neurological examination, evaluation of disability using the expanded disability status scale (EDSS)/Functional System Score (FSS) [7], neuropsychiatric assessment using Beck’s Depression Inventory (BDI) to exclude patients with depression (which could affect cognitive assessment) [8], mental state examination using Mini-Mental State Examination (MMSE) [9], Brief International Cognitive Assessment for Multiple Sclerosis (BICAMS) which focuses on cognitive processing speed, verbal and visual memory which consists of three tests: symbol digits modalities test (SDMT) assessing attention/processing speed/working memory, California verbal learning test-second edition (CVLT-II) assessing learning and verbal memory, and brief visuospatial memory test-revised (BVMT-R) assessing learning and visuospatial memory, and it has been validated in several languages including non-English-speaking countries with Arabic version [10], and neuroradiological assessment with magnetic resonance imaging (MRI) brain and cervical spine with contrast to assess lesion load and presence of atrophy using T1-weighted images, T2-weighted images, and FLAIR images.

Also, they underwent laboratory assessment of CSF level of Chitinase-3-like-1 protein (ng/ml) using ELISA technique (Fig. 1). CSF samples were collected from patients during clinical follow up visits either at time of diagnosis (if they were relapse free > 1 month) or routine follow up (if they were not sampled before). Regarding the control group, they were generally, neurologically examined and underwent cognitive assessment using MMSE and BICMS. CSF samples of the control group were collected during routine procedures, such as spinal anesthesia. Informed written consents were obtained from all individuals who shared in this study and approval of ethical committee of Faculty of Medicine — Cairo University was obtained.

### Statistical analysis

Our data were analyzed using the statistical package for the Social Sciences (SPSS) version 26 (IBM Corp., Armonk, NY, USA). The data was summarized using mean, standard deviation, median, minimum, and maximum in quantitative data and using frequency (count) and relative frequency (percentage) for categorical data. Comparisons between quantitative variables were done using the non-parametric Kruskal-Wallis and Mann-Whitney tests [11]. For comparing categorical data, chi-square (*χ*^2^) test was done. Exact test was used instead when the expected frequency is less than 5 [12]. Correlations between variables were done using Pearson’s correlation coefficient and conducting a two-tailed significance test (*p*-value) [13]. ROC curve was constructed with area under curve analysis performed to detect best cutoff value of CSF CHI3L1 for detection of progression of disease. *p*-values less than 0.05 were considered statistically significant, and all statistical tests were two-tailed.

## Results

### Descriptive results

#### Demographics

Table 2 shows the demographics of the study population. The patient group (*n*=52) had a mean age of 34.85 years (SD = 8.9), with 23.1% male and 76.9% female participants. The control group consisted of 30 individuals with a mean age of 37.91 years (SD = 11.1), of whom 50% was male and 50% was female. Also, educational level (in years) was obtained.

**Table 2:** Demographics of the study population

Items	Patients group (*n* = 52) Mean ± SD	Control group (*n *= 30) Mean ± SD
Age (years)	34.85 ± 8.9	37.91 ± 11.1
Gender		
Male	12 (23.1 %)	15 (50%)
Female	40 (76.9 %)	15 (50%)
Educational levels (years of schooling)	12.2 ± 3.2	11.9 ± 4.u1

#### Clinical characteristics of patients group

Table 3 shows the clinical characteristics of the patient’s group. The mean age of onset of MS was 31.79 years (SD = 7.83), and the mean disease duration was 37.15 months (SD = 32.1). The mean duration from the last relapse was 4.1 months (SD = 2), and the mean number of attacks was 2.71 (SD = 1.6). The mean Expanded Disability Status Scale (EDSS) score was 3.27 (SD = 2.25).

**Table 3:** Clinical characteristics of patient group

Item	Patients group (*n* = 52), mean ± STD
Age of onset (years)	31.79 ± 7.83
Disease duration (months)	37.15 ± 32.1
Duration from last relapse (months)	4.1± 2
Number of attacks	2.71 ± 1.6
Total EDSS	3.27 ± 2.25

Table 4 shows the results of neuropsychiatric tests. Of the 52 patients, 36.6% had severe cognitive impairment, 34.6% had mild cognitive impairment, and 28.8% had no cognitive impairment. Among the affected cases, 57.7% had impaired Symbol Digit Modalities Test (SDMT) scores, 32.7% had impaired California Verbal Learning Test-II (CVLT-II) scores, and 38.5% had impaired Brief Visuospatial Memory Test-Revised (BVMT-R) scores. Table 5 shows the raw mean scores for individual tests of BICAMS and *z*-score for each.

**Table 4:** Neuropsychiatric test assessment

Cognitive test		Count of cases	%
Mini-mental state examination (MMSE)	Severe cognitive impairment (0–9)	19	36.6%
	Mild to moderate cognitive impairment (10–24)	18	34.6%
	No cognitive impairment (25–30)	15	28.8%
BICAMS	SDMT (affected cases)	30	57.7%
	CVLT-II (affected cases)	17	32.7%
	BVMT-R (affected cases)	20	38.5%

**Table 5:** BICAMS tests’ scores in patient and control groups

BICAMS test	Patients group Mean ± SD	Control group Mean ± SD	*z*-score
SDMT	44.2 ± 10.5	55.6 ± 7.8	− 1.46
CVLT-II	41.2 ± 11.6	54.3 ± 7.3	− 1.79
BVMT-R	16.4 ± 4.7	23.7 ± 4.0	− 1.82

#### Radiological characteristics of patients group

Lesion load varied between patients, with a mean lesion number of 10.8 (SD = 3.2). Table 6 shows the other radiological parameters that were studied.

**Table 6:** Radiological characteristics of patient group

		Count	Percent
MRI brain with contrast enhancing lesions	Yes	3	5.8%
	No	49	94.2%
Brain atrophy in MRI	Yes	10	19.2%
	No	42	80.8%
MRI Cervical	Normal	21	40.4%
	Affected	31	59.6%
MRI cervical with contrast enhancing lesions	Yes	0	0.0%
	No	52	100.0%
Cervical cord atrophy in MRI cervical	Yes	17	32.7%
	No	35	67.3%

#### Laboratory assessment of CSF CHI3L1 levels

Table 7 shows the mean CSF CHI3L1 levels for the control group and patient group, as well as for different types of MS. The mean CSF CHI3L1 level was significantly higher in the patient group (165.2 ng/ml, SD = 77.66) than in the control group (28.46 ng/ml, SD = 10.96). The mean CSF CHI3L1 levels for the different types of MS were as follows: 107.37 ng/ml (SD = 19.23) for relapsing-remitting MS (RRMS), 244.27 ng/ml (SD = 52.99) for progressive MS (PMS), 256.00 ng/ml (SD = 70.50) for primary progressive MS (PPMS), and 239.88 ng/ml (SD = 46.90) for secondary progressive MS (SPMS).

**Table 7:** Laboratory assessment of CSF CHI3L1 levels

Group (*n*)	Mean CSF CHI3L1 ± SD (ng/ml)
Control group (35)	28.46 ± 10.96
Patients group (52)	165.2 ± 77.66
RRMS (30)	107.37 ± 19.23
Progressive MS (PMS) (22)	244.27 ± 52.99
PPMS (6)	256.00 ± 70.50
SPMS (16)	239.88 ± 46.90

### Comparative results

#### Comparison between MS cases and control in CSF CHI3L1 levels

Table 8 shows the comparison of CSF CHI3L1 levels between the patient group and the control group, as well as Tables 9 and 10 which show the comparison of CSF CHI3L1 levels between different subtypes of MS. The differences were statistically significant for all comparisons except for the comparison between PPMS and SPMS.

**Table 8:** Comparison between MS cases and control

	Patients group		Control group		*p* value
	Mean	SD	Mean	SD	
CSF CHI3L1 level ng/ml	165.29	77.66	28.46	10.96	< 0.001

**Table 9:** Comparison between RRMS and PMS

	RRMS		PMS		*p* value
	Mean	SD	Mean	SD	
CSF CHI3L1 level ng/ml	107.37	19.23	244.27	52.99	< 0.001

**Table 10:** Comparison between PPMS and SPMS

	PPMS		SPMS		*p* value
	Mean	SD	Mean	SD	
CSF CHI3L1 level ng/ml	256.00	70.50	239.88	46.90	0.802

Comparison between RRMS and PMS in CSF CHI3L1 levels

Comparison between PPMS and SPMS in CSF CHI3L1 levels

**Fig. 2:**
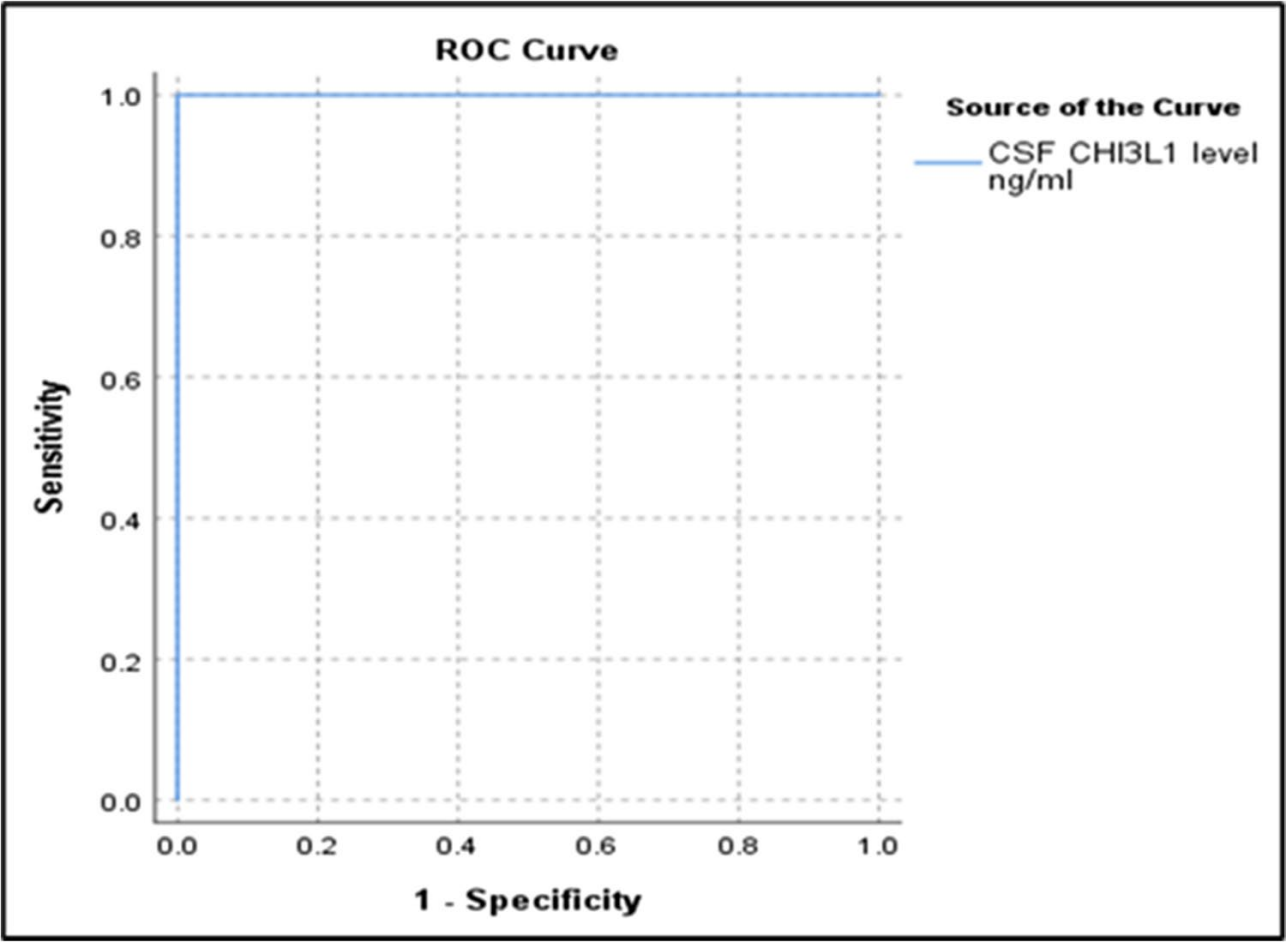
Cut-off value in CSF level of CSF CHI3L1 between RRMS and PMS

Cut-off value in CSF level of CSF CHI3L1 between RRMS and PMS by using ROC curve = 154 ng/ml; Fig. 2

### Correlative results

Correlations were measured between all studied parameters and CSF CHI3L1 level, but only significant correlations were reported here.

#### Correlation between CSF level of CHI3L1 and different clinical parameters

There was a highly statistically significant good positive correlation between duration of the disease and CSF level of CHI3L1 (*p* > 0.001, *r* = 0.608).

Also, there was a statistically significant good positive correlation between number of attacks and CSF level of CHI3L1 (*p* = 0.001, *r* = 0.460).

In addition, there was a highly statistically significant good positive correlation between EDSS and CSF level of CHI3L1 (*p* > 0.001, *r* = 0.730).

The severity of cognitive impairment (either MMSE or BICAMS) showed significant good positive correlation with CSF CHI3L1 level (*p* = 0.001, *r* = 0.670, 0.563 consecutively). Episodic memory was the most correlated cognitive domain with CSF CHI3L1 level.

#### Correlation between CSF level of CHI3L1 and radiological parameters

There was a highly statistically significant good positive correlation between T2 lesion number and CSF level of CHI3L1 (*p* > 0.001, *r* = 0.601).

Also, there was a highly statistically significant good positive correlation between presence of MRI brain atrophy and CSF level of CHI3L1 (*p* > 0.001, *r* = 0.581).

In addition, there was a highly statistically significant good positive correlation between presence of MRI brain black holes and CSF level of CHI3L1 (*p* > 0.001, *r* = 0.531).

In MRI CS, there was statistically significant positive correlation with presence of demyelinating lesions, cord atrophy, and CSF level of CHI3L1 level (*p* = 0.001, *r* = 0.520, 0.543 consecutively).

## Discussion

Within CNS, CHI3L1 is mainly secreted by reactive protoplasmic astrocytes, and primed microglial cells in lesions with low inflammatory activity. CHI3L1 could be measured in serum and CSF, where increase throughout MS stages seems to be derived from the extension of diffuse brain inflammation associated with neurologic impairment rather than acute inflammation during relapses and the detrimental role of astrocyte activation in disease pathogenesis [3]. There are several studies in the literature that have compared ELISA with other more sensitive methods for measuring CSF CHI3L1 levels. For example, a study by Disanto and colleagues in 2017 that compared the performance of ELISA and SIMOA for measuring CSF CHI3L1 levels in patients with multiple sclerosis. The authors found that the two methods were highly correlated (*r* = 0.92), but SIMOA was more sensitive than ELISA, detecting CHI3L1 in samples that were negative by ELISA [14]. Another study by Burman and colleagues in 2018 compared the performance of ELISA, SIMOA, and Luminex xMAP for measuring CSF CHI3L1 levels in patients with Alzheimer’s disease. The authors found that all three methods were highly correlated (*r* > 0.8), but SIMOA was the most sensitive, followed by Luminex and ELISA [15]. A study by Semra and colleagues in 2003 compared the performance of ELISA and Western blot for measuring CSF CHI3L1 levels in patients with multiple sclerosis. The authors found that ELISA had a lower detection limit than Western blot, but the two methods were highly correlated (*r* = 0.85) [16]. In our study, we were mainly concerned with measuring CSF level of CHI3L1 in MS patients and comparing its level with normal control individuals. Also comparing its level between subtypes of MS. In addition, we aimed to study the relationship between the level of CHI3L1 in CSF and the clinical disability parameters in MS, including cognitive impairment with the aim of determining whether CHI3L1 could be used as a biomarker for MS progression. We tried to obtain a cut-off point of CSF CHI3L1 level that can help to differentiate between different MS types (Fig. 2). Our results showed that there was a significant increase in CSF level of CHI3L1 in MS patients than normal control. Also, there was a significant increase of CSF CHI3L1 level in MS subgroups compared to controls and significant increase of CSF CHI3L1 level in PPMS and SPMS compared to RRMS. In agreement with our results, Soelberg and colleagues in 2017 confirmed that CSF levels of CHI3L1 were higher in MS patients compared to non-MS controls [17]. Rucsanda Pinteac and colleagues in 2021 explained that as within CNS lesions of MS patients, CHI3L1 expression was present both in macrophages/microglial cells and astrocytes, with an important contribution of protoplasmic astrocytes to CHI3L1 expression as a result of active chronic lesions classified as having high degree of chronic inflammatory activity, reactive gliosis, and axonal loss [2]. Moreover, Cubas-Nuñez and colleagues in 2021 demonstrated that discrepancies between RRMS and progressive MS may be due to the location of lesions and/or differences in BBB permeability [3]. Regarding lesion location, PMS had higher cortical and spinal cord lesion load than RRMS. In this context, histological studies clarified that protoplasmic astrocytes are predominant in cortical gray matter and spinal cord more than in white matter, so this could explain the increasing level of CHI3L1 in PMS [18, 19]. In this regard, Sanfilippo and colleagues in 2017 confirmed that CHI3L1 is produced from activation of microglia and astrocytes during the progression of neurodegeneration in cortex and in the spinal cord of neurodegenerative diseases like MS, ALS, and Alzheimer’s disease [20]. Regarding differences in BBB permeability in relapsing MS, there are inflammatory infiltrates with focal breakdown of the blood-brain barrier due to tight junction (TJ) pathology, lymphocyte infiltration, and upregulation of proinflammatory cytokines and chemokines such as IL-1β, IL-17, IL-22, IFN-γ, and CCL2. Most of these immune mediators are released by leukocytes during transmigration, and within the CNS and aside from promoting and expanding immune cell activation, they can also affect the integrity of the BBB. In vivo and in vitro studies have shown that IL-1β indirectly destabilizes the BBB by inducing expression of matrix metalloproteinase-9 (MMP-9), an enzyme known to cleave occludin, ZO-1, claudin-5, and other junctional complex associated molecules. Degradation/downregulation of TJ proteins correlate with the elevated levels of MMP-9 expression reported in MS patients. IL-17 and IL-22 have also been associated with changes in the BBB. In this regard, treatment of primary cultures of human BBB-endothelial cells with both cytokines induced alterations in the BBB permeability which are the main drivers of immunemediated demyelination and axonal transection through formation of new lesions [21]. While active lymphocytic lesions are common in relapsing MS, in progressive disease, both non-lymphocytic chronic active and chronic inactive lesions and active neurodegeneration are predominant. In progressive MS, inflammation likely continues to drive injury by astrocytes and microglia which are the main source of CHI3L1 in CNS, but this inflammation typically occurs in the setting of an intact blood-brain barrier. These discrepancies suggest a different pattern of release of inflammatory cells that may be related to the lesion location or to the diffuse inflammatory changes representative of the progressive disease. The relationship between CHI3L1 CSF and active neuroinflammation, and the elevation of CHI3L1 CSF in PMS, implies a possible relationship between this biomarker and the progression of disability [22, 23]. Furthermore, we found that the cut off value in CSF CHI3L1 between RRMS and progressive MS was 154 ng/ml, and our results are near to these of other studies as that of Modvig and colleagues in 2015 in which the cut off value for progression was 147 ng/ml. But there was no significant difference between PPMS and SPMS in CHI3L1 level to get cut off value [24]. This can give a very important clue for the proper choice of DMD and the time to escalate DMDs during the course of the disease. Escalation should be done in RRMS patients with high levels of CSF CHI3L1 before reaching cut off point level, to protect multiple sclerosis patient from further progression and disability [25]. Our results showed that there was a highly significant positive correlation between CSF level of CHI3L1 and duration of the disease. Kušnierová and colleagues in 2020 suggested that increased levels of CSF CHI3L1 in patients with CNS inflammation compared with healthy individuals, and its increase with increasing age is consistent with the hypothesis that lower-grade inflammatory processes are induced in the aging brain [26]. Also, Sellebjerg and colleagues in 2019 and Gil-Perotin and colleagues in 2019 confirmed that CSF CHI3L1 is very dependent on the duration of the disease; patients with progressive disease are usually older than RRMS patients with more disease duration [27, 28]. Also, the abnormal reactivation of CNS astrocytes and priming of microglia (during which the cell body hypertrophies and its processes shorten and branch extensively and primed microglia respond much vigorously to brain injury, inflammation and aging challenge, and boost the activation by switching from an anti-inflammation, potentially protective phenotype to a pro-inflammation destructive phenotype) lead to gliosis and atrophic changes that occur in older ages in MS patients. This was explained by Yeo and colleagues in 2019 as MS patients could have continuous subclinical neurodegeneration and chronic inflammatory process in spite of there is no obviously clinical relapses [29]. Our study showed that there was significant correlation between CSF CHI3L1 and number of attacks, and this was explained by Sellebjerg and colleagues in 2019 who demonstrated that in RRMS, every single attack was associated with astrocytes and axonal damage then followed by gliosis and priming of CNS astrocytes and microglia so CSF CHI3L1 was increased as a result to that damage [27]. Our study showed that there was significant good positive correlation between CSF level of CHI3L1 and EDSS, and that was confirmed by lots of previous studies as Gil-Perotin and colleagues 2019, and Pérez-Miralles and colleagues in 2020, who demonstrated that higher EDSS was associated with higher CHI3L1 levels which confirms its role with disability progression [28, 30]. Interestingly, CSF CHI3L1 levels were associated with the development of brain atrophy evaluated by the brain parenchymal fraction change as well as cervical cord atrophy, both leading to increase disability in MS patients [31, 32]. Also, Burman and colleagues in 2016 suggested that increased levels of CSF CHI3L1 are associated with tissue damage related to inflammation and might predict residual disabilities and possibly cumulative damage over time, which were found more in progressive MS patients than RRMS, so high levels of CHI3L1 were associated with higher EDSS scores [33]. Cognitive impairment is a common and often debilitating feature of multiple sclerosis (MS). The pathophysiology of cognitive impairment in multiple sclerosis (MS) is complex and multifactorial, involving both structural and functional changes in the central nervous system (CNS), as well as inflammatory and neurodegenerative processes [34]. One potential mechanism underlying cognitive impairment in MS is the breakdown of the blood-brain barrier (BBB), which can lead to the infiltration of immune cells into the CNS and subsequent inflammation and demyelination. This process can disrupt neural circuits and impair cognitive function. Another potential mechanism is neurodegeneration, which can occur in the absence of active inflammation and can result in the loss of axons, neurons, and synapses. Neurodegeneration has been linked to cognitive impairment in MS, particularly in the domains of information processing speed and memory [35]. CHI3L1, a marker of inflammation and neurodegeneration, has been implicated in the pathophysiology of cognitive impairment in MS. Recent studies have suggested that CSF biomarkers, such as CHI3L1, may be useful in predicting cognitive impairment in MS [36]. Our study showed significant cognitive impairment associated with higher levels of CSF CHI3L1. Multiple studies investigate the relation between CSF concentration of chitinase-3-like-1 proteins and cognitive impairment which seems to imply that glial activation which is involved in atrophic changes of the brain and axonal loss may negatively influence different cognitive domains [37, 38]. A study by Arrambide and colleagues in 2015 found that CSF CHI3L1 levels were significantly higher in MS patients with cognitive impairment compared to those without, and that higher CHI3L1 levels were associated with more severe cognitive impairment, particularly in the domains of verbal memory and attention [39]. This goes in line with the findings of other studies investigating the relationship between CSF CHI3L1 levels and cognitive impairment in MS patients as in the study of Semra and colleagues in 2003, which concluded that the presence of high levels of CHI3L1 in the CSF and serum of MS patients correlated with more severe disease and a greater number of relapses [16]. Also, the study by Liu and colleagues in 2022 aimed to review the current understanding of the role of antibodies and inflammatory agents as CHI3L1 in the pathophysiology of MS, their clinical relevance, and their potential as biomarkers for the disease. It concluded that higher levels of CHI3L1 in serum were associated with more severe cognitive impairment and more disease progression [40]. Moreover, the literature suggests that glial activation, which is involved in atrophic changes of the brain and axonal loss, may negatively influence different cognitive domains (e.g., episodic memory, processing speed, executive function) in MS patients [41]. Overall, these studies suggest that CSF CHI3L1 levels may be a useful biomarker for predicting cognitive impairment severity in MS, particularly in the domains of information processing speed, working memory, verbal memory, and attention. Regarding radiological data, our results showed that the patients with higher levels of CSF CHI3L1 showed higher T2 lesion number in MRI brain and that goes in concordance with Kutzelnigg and colleagues in 2005 and Cubas-Núñez and colleagues in 2021 results [3, 42]. There was no relation between number of gadolinium-enhancing lesions (GEL) and levels of CSF CHI3L1 that was matching with Quintana and colleagues in 2018, who did not find an association between CSF CHI3L1 levels and presence of GEL, nor increasing numbers or size of GEL [38]. This was explained by that during relapse, inflamed areas, which are also associated with blood-brain barrier breakdown, appear as gadolinium-enhancing lesions on MRI around the time they develop; this is not associated with the progressive phase in which activated astrocytes and microglia secret CHI3L1. We also found that there was a highly significant good positive correlation between presence MRI brain black holes and brain atrophy and CSF level of CHI3L1, which might be explained by the diffuse neurodegeneration and axonal loss along with ongoing astrocytic and microglial activation results in higher levels of CSF CHI3L1 in progressive MS, which was confirmed by many previous studies [31, 38]. Also, we found that higher levels of CSF CHI3L1 had a significant positive correlation with cervical MRI lesion number and cervical cord atrophy, which was confirmed and explained by a very recent study of Ruth and colleagues in 2021. Also, spinal cord atrophy in MS correlates with clinical disability and disease progression [31]. A possible explanation by Ruth Pinteac and colleagues was that progressive MS is a neurodegenerative rather than inflammatory process which is the main driver of disease progression. Neurodegeneration generates higher relative volume losses in the spinal cord than in the brain due to a lack of compensatory capabilities in this relatively small structure and diffuse neurodegeneration along with ongoing astrocytic and microglial activation results in higher levels of CSF CHI3L1 in progressive MS, which correlates with cervical spinal cord volume reduction [2].

## Conclusion

Based on the available evidence, it can be concluded that CHI3L1 is a promising biomarker of disease progression in multiple sclerosis (MS). Studies have shown that the levels of CHI3L1 in the cerebrospinal fluid (CSF) are elevated in patients with progressive MS compared to those with relapsing-remitting MS (RRMS). Moreover, higher CSF levels of CHI3L1 are associated with greater disability, including motor, cognitive, and radiological aspects of the disease. These findings suggest that CHI3L1 could be a useful tool for monitoring disease progression and predicting outcomes in patients with MS. However, further research is needed to fully establish the clinical utility of CHI3L1 as a biomarker in MS.
